# The Impact of Protein Supplementation Targeted at Improving Muscle Mass on Strength in Cancer Patients: A Scoping Review

**DOI:** 10.3390/nu12072099

**Published:** 2020-07-16

**Authors:** Michela Zanetti, Gianluca Gortan Cappellari, Rocco Barazzoni, Gianfranco Sanson

**Affiliations:** Department of Medical, Surgical and Health Sciences, University of Trieste, 34127 Trieste, Italy; gigici2@iol.it (G.G.C.); barazzon@units.it (R.B.); gsanson@units.it (G.S.)

**Keywords:** amino acids supplementation, cancer, handgrip, muscle strength, protein supplementation, scoping review

## Abstract

Deterioration of muscle strength during cancer results in functional limitation, poor quality of life and reduced survival. The indirect effects on muscle strength of nutritional interventions based on protein and amino acid derivatives targeted at improving muscle mass are poorly documented. A scoping review was performed to examine the available evidence on the effects of proteins, amino acids and their derivatives on muscle strength in adult cancer patients. Pubmed and Scopus databases were searched to identify research articles published in the last 10 years. Fourteen studies met the inclusion criteria, showing that changes in muscle strength following protein or amino acid supplementation are generally concordant with those in muscle mass in cancer patients. Administration of both energy and proteins in the presence of reduced oral intakes results in more robust effects on both muscle strength and mass. It is not clear whether this is due to the correction of the energy deficit or to an interaction between proteins and other macronutrients. The optimal mixture, type, and dose of amino acid/protein supplementation alone or in combination with other anabolic strategies should be determined to provide the best nutritional approach in cancer.

## 1. Introduction

Sarcopenia is a well-recognized feature of cancer and it is notably associated with poor clinical outcomes, including increased rate of postoperative infections, delayed recovery after surgery [1], higher risk of treatment toxicity [2,3,4], reduced progression-free and overall survival [5,6,7], and impaired quality of life (QoL) [8]. Depending on cancer type and stage, activation of proteolytic pathways results in early loss of skeletal muscle, which can further progress to depletion of both fat and muscle mass, constituting the condition of cachexia [9]. In relation to cancer, cachexia is described as a loss of muscle mass (alone or associated with concomitant loss of fat mass) which cannot be counteracted by nutritional support, resulting in progressive functional impairment [9]. Weight loss >5% over six months or weight loss >2% combined with either BMI <20 or sarcopenia defines cachexia. According to the same international consensus [9] sarcopenia can be diagnosed in the presence of reduced skeletal muscle mass (generally below the 5th percentile). Body composition measurements for which standardized cut offs are identified for the diagnosis of sarcopenia include mid-upper arm area by anthropometry [10], appendicular skeletal muscle index by dual X-ray absorptiometry (DXA) [11], skeletal muscle index by computed tomography (CT) [6,7], and fat-free mass index by bioimpedance analysis (BIA) [12]. Low muscle mass is closely associated with poor short- and long-term clinical outcomes in cancer patients, including increased rate of surgical complications, chemotherapy-related toxicity, and reduced survival [3,13], and a predictive value of reduced skeletal muscularity in cancer-related morbidity and mortality has been convincingly demonstrated by many studies [6,13]. Further investigations have shown that low muscle radiation attenuation as assessed by computed tomography is associated with fat infiltration, and this phenomenon is linked with poor prognosis in some types of cancers [14,15]; fat infiltration (also known as myosteatosis) has therefore been intensively investigated in an effort to improve the prognostic accuracy of altered body composition during cancer and related therapies [16]. Although low muscle strength is recognized as a key component of a patient’s functional status and QoL and is a potential therapeutic target [17], standardized cut offs for low muscle strength have not yet been identified in the definition of cancer-associated cachexia and sarcopenia [9]. This is especially important when considering the growing incidence of cancer with aging [18] and the declining mortality and increasing survival rates of many cancer types through programs revolving around effective antineoplastic treatment [19]. Sarcopenia, which is common among older adults, is a condition of muscle failure characterized by both reduced muscle strength and muscle quality/quantity; however, in the most recent geriatric definition of sarcopenia, muscle strength is the key characteristic of the condition [20]; handgrip strength is the best assessment technique because of its ease of use and its correlation with clinical outcomes [20]. As demonstrated by many studies, nutritional interventions may reverse loss of muscle mass and therefore have the potential to increase treatment tolerability and reduce complications [21] but whether increased muscle mass also translates into better strength is less clear.

As a matter of fact, low muscle strength (or dynapenia) can also be found without low muscle mass (myopenia) [22]; in the presence of cancer-associated muscle loss, the rate of concordance between dynapenia and myopenia is fair [23], highlighting the need to separately assess both muscle mass and strength in each patient. It should also be noted that while in some groups of individuals, e.g., elderly cancer patients, it may be almost impossible to separate the two conditions, in younger patients the prevalence of sarcopenia and dynapenia may depend on cancer type [24] and treatment. This is especially true when considering therapies that notably affect muscle mass and strength, such as hormone therapy [25]. From a clinical standpoint, dynapenia is very relevant to daily practice, as low muscle strength predicts poor physical performance and sarcopenia-related outcomes, including disability and mortality. Handgrip strength is directly associated with cancer-related fatigue, poor QoL, chemotherapy-induced toxicity, postoperative complications, length of hospital stay, and mortality in cancer patients [17,26,27,28,29,30,31]. In addition, while muscle quantity and quality are technically more difficult to assess, muscle strength is detectable even in daily practice; therefore, targeting muscle strength may represent an appealing approach to improve functionality and QoL in cancer patients. Whether nutritional strategies targeted at recovering muscle mass also positively impact on muscle strength and whether this is associated with improved clinical outcomes, namely functional capacity, treatment tolerability, and survival, is currently unknown. 

Among nutritional strategies, adequate protein and amino acid supply are required for optimal protein anabolism in the setting of cancer-associated loss of muscle mass and strength. 

This scoping review focuses on the most recent available evidence for the effects of nutritional interventions based on proteins, amino acids, and their derivatives in cancer patients at different stages of the disease in order to address the question of whether there is therapeutic potential regarding protein supplementation to maintain/improve not only muscle mass but also strength in order to reduce morbidity and adverse clinical outcomes in cancer.

## 2. Methods 

A scoping review was chosen as it represents an ideal tool to examine the availability of studies on a given topic not previously extensively reviewed, and it is generally heterogeneous. Different from systematic reviews, a scoping review does not perform a critical assessment of methodological limitations or risk of bias of the evidence included, but aims to provide an overview of existing studies, reporting their focuses and identifying gaps in the knowledge base [32]. Also, while systematic reviews are focused on very specific questions which are answered by a selection of a small number of studies whose quality is carefully assessed, scoping reviews address broader topics and the quality of the included studies is not scrutinized [33].

The study was conducted according to the Preferred Reporting Items for Systematic Reviews and Meta-analysis—Extension for Scoping Reviews (PRISMA-ScR) checklist [34].

### 2.1. Eligibility Criteria

The review included studies comparing treatment with amino acids or proteins or other protein-derived supplements in cancer patients. Studies comparing different protein-derived supplements were also included. Studies were included if they considered muscle strength as an outcome measure. Publications were excluded if the association between protein supplementation and muscle strength was not specifically examined or if data about such analyses were not clearly reported. Studies reporting data on pediatric or noncancer cohorts and research conducted on animal models were excluded as well.

### 2.2. Search Strategy

In order to ensure a comprehensive search of the literature, the electronic search was conducted in the PubMed and Scopus databases according to the study aim by combining a wide series of potentially relevant terms using Boolean operators. Keywords focused on muscle strength, cancer, and different types of nutrition treatment based on protein and amino acids; search terms regarding the types of studies or outcomes were not included to avoid restricting the search. As the aim of this review was focused on the available most recent evidence, the search was restricted to primary research articles published in the last 10 years (i.e., from 2010 to 2020), also excluding papers not published in English. Table 1 shows the final search strings run in the selected databases. The latest search was performed on 31 March 2020.

### 2.3. Study Selection

After completing the database searches, citations were compiled and entered into EndNote X7.1, where duplicates of articles found in both databases were removed. Then, two authors (GS and MZ) independently scrutinized the records throughout two stages. In the first stage, potentially pertinent articles were screened based on the respective titles and abstracts; studies devoid of abstracts were excluded without any further consideration. After a comparison based on a consensus, the full texts of documents were obtained for the selected abstracts. In the second stage, the selected manuscripts were independently assessed by the two above authors, and those judged to be pertinent were subsequently discussed in detail to achieve a consensus. In the case of disagreement, a third author (GGC or RB) was consulted.

### 2.4. Synthesis of Results

Two authors (GS and MZ) extracted the data using a standard data extraction form and reviewed data from each included trial. Information on study design, study size by patient number, setting, study limitations, patient characteristics, outcome measures, and results were collected and evaluated. Data were checked by a third reviewer (GGC or RB) to ensure accuracy. Following data extraction, a narrative synthesis was provided to summarize the main results of the studies in light of the review objectives. This approach allowed the current evidence to be synthetized and gaps and opportunities for future research to be identified. 

## 3. Results

**Figure 1:**
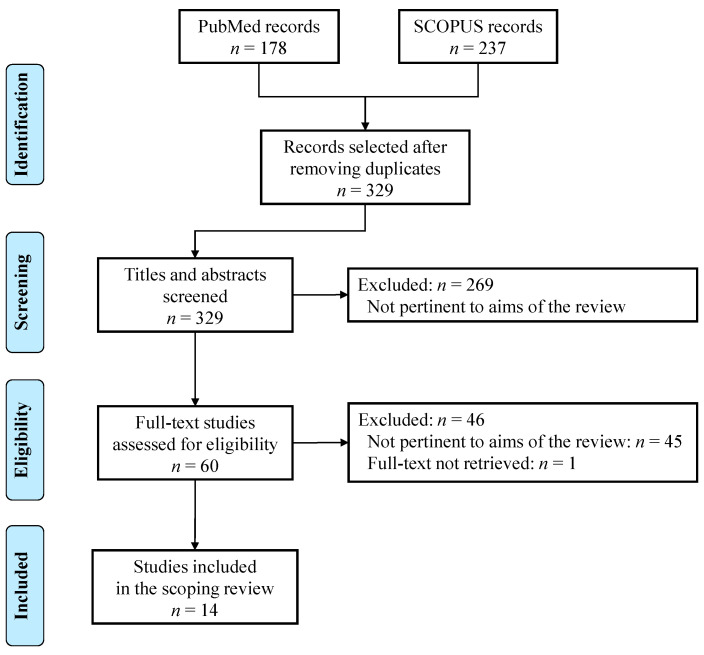
Flow diagram of the studies selection process.

A total of 329 potentially relevant articles were identified after removing duplicates. Among the 60 full text documents assessed for inclusion, 14 were judged as pertinent and included in the scoping review (Figure 1). 

### 3.1. Trials with Amino Acids, Proteins, and Protein-Derived Dietary Supplements

In a randomized controlled trial of 166 malnourished advanced cancer patients with mixed tumors undergoing chemotherapy, Cereda et al. tested the effect of nutritional counseling with or without whey protein isolate (WPI) supplementation (20 g/day) for three months and showed a more favourable evolution in the group treated with counseling plus WPI as compared with patients treated with counseling alone with improved bioimpedance parameters (phase angle-PhA and fat-free mass index-FFMI) and muscle strength, and a reduced risk of chemotherapy toxicity [35]. Similar results were shown by Madeddu et al. who, in an uncontrolled trial including 25 cachectic stage IV patients with BMI < 20 kg/m^2^ and tumors at different sites assessed the effects of mixed amino acid supplementation for eight weeks on several muscle (including lean body mass and grip strength), quality of life (fatigue), and laboratory parameters (albumin, proinflammatory cytokines, and markers of oxidative stress) [36]. No nutritional data regarding energy and protein requirements and intake were reported. At the end of the study period, increased muscle strength and total serum albumin, as well as decreased reactive oxygen species (ROS), following the intervention were demonstrated. WPI (50 g/day) alone or in combination with resistance training (three sessions/week, ~50 min in duration for 12 weeks) were administered to 37 prostate cancer patients on androgen deprivation therapy. The effects of combined WPI supplementation and resistance activity on sarcopenia, muscle strength, and QoL were compared to those of single interventions (resistance training or protein supplementation) or control stretching. While resistance training resulted in improved sarcopenia, muscle strength, and QoL, no effects of protein administration were observed on any of the explored outcomes after the 12-week intervention [37]. Similar results were obtained by Lonbro et al. [38], who randomized thirty head and neck cancer patients treated with radiotherapy alone or associated with chemotherapy/immunotherapy into resistance training (30 training sessions evenly dispersed over the study period) or resistance training plus creatine (5 g) and protein (30 g) groups for 12 weeks. At the end of the study period, muscle strength, lean body mass, and functional performance increased to the same extent in both groups. Thus, this study confirms that addition of protein supplementation to resistance training does not result in any additional benefit in terms of muscle strength and mass as compared with resistance activity alone in cancer patients. In a small study of 33 breast cancer survivors, Madzima et al. [39] tested the effects of a 12-week program of resistance training alone (two sessions/week, chest press and leg extension, one repetition maximum) or in combination with whey/casein protein isolate (40 g/day) on muscle strength, body composition, and serum concentrations of selected adipokines and inflammatory markers. Both interventions increased muscle performance, body composition, and plasma levels of insulin-like growth factor 1, without any additional effect of supplementary protein. 

However, in this trial the effects of protein intervention may have been underestimated due to a spontaneous reduction in dietary protein intake from oral food, resulting in a net increase in daily protein intake of 17 g. Another study by Mantovani et al. [40] investigated the effect of L-carnitine 3 g/day for four months in 332 patients with advanced stage tumors at any site and cancer-related anorexia/cachexia syndrome on a wide array of functional and anatomic outcomes, including grip strength, QoL, serum levels of interleukin 6 (IL-6), tumor necrosis factor α (TNFα), ROS, Glasgow Prognostic Score (GPS), total daily physical activity, and performance status as secondary outcomes. Primary endpoints were increase in lean body mass and decrease in fatigue and resting energy expenditure (REE). The participants were given either L-carnitine alone or in combination with medroxyprogesterone/megestrol acetate, oral eicosapentaenoic acid (EPA), and thalidomide. The most effective treatment for the primary endpoints of lean body mass, REE, and fatigue and for the secondary endpoints of grip strength, GPS, and performance status was the combination regimen that included all agents; no effect was demonstrated by L-carnitine supplementation alone. The effects of supplementation with soy/whey proteins plus a natural diet versus the natural diet alone were assessed in 24 patients with acute leukemia. Nutritional intervention started 30 days before transplantation and lasted for 30 days after the transplantation phase. The endpoint measurements included biochemical variables (total protein, albumin, and globulin levels) and anthropometric and functional parameters (body weight, body mass index and circumferences, triceps skin-fold thickness, muscle strength). Despite significant reductions in energy and protein intakes after transplantation in both groups, patients supplemented with soy/whey proteins demonstrated increased muscle strength, mass, and serum proteins [41]. Interestingly, nutritional intervention was associated with significantly shortened stem cell engraftment time. Finally, in another study, [42] perioperative supplementation with HMB/Arg/Gln (15.2 g/day) was investigated in 60 patients undergoing open surgery for abdominal cancer. The supplementation was provided for three days preoperatively and for seven days postoperatively. Muscle strength was included among the secondary endpoints, along with postoperative duration of hospital stay, total-body skeletal muscle mass, skin water content, and the incidence of complications other than wound recovery (primary endpoint). The results showed no significant effect of beta-hydroxy-beta-methylbutyrate/arginine/glutamine (HMB/Arg/Gln) administration on handgrip strength and body composition in the active treatment group compared with the placebo. No significant differences were demonstrated in the incidence of other complications, body composition, or skin water content between the two groups. Main characteristics and conclusions of referenced studies are reported in Table 2.

### 3.2. Trials with Nutritional Supplementation Including Proteins and Other Macronutrients

Six studies looking at calorie and protein provision via oral nutritional supplementation or enteral/parenteral nutrition were identified (Table 2). All of these studies investigated patients with advanced tumors or who were obviously undernourished. Throughout the studies, no methodology allowed for the differentiation of the effects of protein supplementation from those of calorie or of other macronutrients on functional muscle outcomes.

The effects of oral nutritional supplements (ONS) were examined in two studies. ONS were administered for three months to severely undernourished cancer patients or those at high risk of malnutrition as part of an individualized nutritional intervention (counseling plus food fortification and ONS if required), resulting in improved energy and protein intake but no impact on nutritional status, muscle strength, physical functioning, or QoL as compared to the control group, who received usual care without specific nutritional intervention [48]. On the other hand, Cereda et al. [45] showed that –in 159 head and neck cancer patients undergoing RT or RT plus systemic treatment– administration of an oral formula providing 500 kcal and 23 g of protein daily prevented weight loss (primary endpoint) but did not significantly improve muscle mass as estimated by phase angle and bioimpedance analysis; however, there was a trend (*p* = 0.057) toward improved muscle strength and significant amelioration of QoL and anticancer treatment tolerance (systemic treatment dose reduction or complete suspension) (secondary endpoints). In another study including 20 head and neck cancer patients undergoing chemoradiotherapy [43], dietetic counseling alone or associated with supplementation by enteral nutrition for three months was not able to prevent loss of muscle strength and body weight. No correlation was found between parameters of nutritional status, muscle mass and strength, and survival. In contrast, in a nonrandomized trial, 12 weeks of nutritional intervention by ONS/EN/PN significantly increased handgrip strength and walking distance in 82 mixed advanced solid tumor patients undergoing anticancer therapy, the latter effect being further magnified by an intervention based on electrostimulation and a physical activity program (24 trainings with seven different dynamic exercises progressively increasing in duration from 12 min/session to 20 min/session). The intervention (but not the control) group also showed significant changes in muscle mass and body composition, while no significant changes were detected in QoL, fatigue, and laboratory variables (total plasma albumin and C-reactive protein) [47]. 

Two studies examined the effects of short- (7 days) [44] and long-term (24 weeks) [46] parenteral nutrition in situations where oral/enteral nutrition was contraindicated. Caccialanza et al. [44] examined changes in phase angle, handgrip strength, and prealbumin levels in 131 hypophagic hospitalized patients at nutritional risk, mainly in the advanced disease stage, while Obling et al. [46] measured variations in bioimpedance analysis-estimated fat-free mass, muscle strength, quality of life, and survival in 47 malnourished outpatients with incurable gastrointestinal cancer. Both studies showed improved muscle strength and mass after the intervention. Significant increases in prealbumin plasma levels were detected [44], along with improved QoL [46]. No changes in hydration status, metabolic complications, or survival were observed [44,46].

## 4. Discussion

In this scoping review, we assessed the clinical effectiveness of nutritional interventions based on protein supply alone or in combination with other macronutrients and primarily targeted at muscle mass on muscle strength in cancer patients. Although many studies were found that addressed this issue, very few fulfilled the inclusion criteria selected for this review. The number of included patients was variable, as were the nutritional treatments used in the study and other associated interventions (physical activity programs). Overall, the lack of evidence from high-quality studies did not allow us to draw any definitive conclusion regarding the effect of protein-based interventions on muscle strength in cancer. However, some general indications could be extrapolated from the available evidence.

The spectrum of alterations in muscle mass and strength in cancer is complex and multifaceted. Assessment of muscle mass and quality progressively gained more attention due to the prognostic implications associated with muscle wasting or myopenia, including treatment-related toxicity, tumor progression, and reduced overall survival [7,49,50,51,52]. However, from a functional perspective, muscle strength is at least as relevant as mass, since dynapenia, which is consistently reported in cancer patients [53], has profound consequences and long-term impact on QoL, including performance limitations and restricted ability to conduct routine activities [54,55]. Additionally, previous studies have shown that skeletal muscle mass is not linearly associated with impaired physical function and performance in the elderly with cancer [53], therefore, no conclusions on physical strength could be extrapolated from data regarding muscle mass. Finally, the effects of nutritional interventions targeted at enhancing muscle mass on strength were poorly explored. The assumption that improving skeletal muscle mass also impacts strength should not be taken for granted; as a matter of fact, previous studies demonstrated that changes in lean mass only explain 5% of variability of strength decline in the elderly [56]. In cancer patients, although a good correlation was observed in basal conditions between muscle strength and mass, in the presence of loss of muscle mass this association is weaker [23]. The current poor understanding of the relationship between changes in muscle mass and strength during cancer is highlighted by the fact that muscle strength is often included among the secondary outcomes of research studies, with quantity of lean mass generally being the primary outcome. In addition, the effects of protein-based nutritional interventions to enhance muscle strength maintenance/recovery and their correlation with muscle mass were scarcely investigated, and filling this gap is a key step toward the prevention of cancer-induced sarcopenia and cachexia. 

The results of interventions with amino acids, proteins, and their derivatives on muscle strength are contradictory, with some studies showing improvements [35,36,42] not confirmed by others [37,41]. Evaluation of the effects of modular supplementation with amino acids/proteins is difficult to perform in active oncologic patients, as biases can result from methodological issues related to incomplete coverage of energy requirements by spontaneous feeding [35]. In this case, results should be interpreted with extreme caution, as positive findings could be linked more to the fulfillment of the energy target rather than to specific effects of proteins on muscle strength, especially in very undernourished/cachectic patients [36]. Data from a different cohort of cancer patients at low risk of malnutrition did not show any effects of protein supplementation up to 50 g/day on muscle strength [37]. However, it should be noted that, despite the low risk of malnutrition, this population underwent androgen deprivation therapy, which notably exerts profound adverse effects on muscle mass with less clear effects on muscle strength [25]. Overall, it seems that when anabolic/anticatabolic interventions are applied alone or in association with dietary proteins, their effects overcome those of protein supplementation alone [37,38,39], without any additional synergistic contribution due to the addition of dietary proteins. In spite of the small sample size and the heterogeneity of patients included in the studies that explored these mixed interventions, the conclusions were quite clear. Similar results were shown associating protein-based interventions with anti-inflammatory/orexigenic pharmacologic treatments [40].

Studies on patients at high risk of malnutrition or who were obviously malnourished supplemented with high-energy ONS to contrast both calorie and protein deficits provided a clearer picture [45,48], showing no [48] or borderline significant effects [45] of energy and protein supplementation on muscle strength, although other relevant parameters, including dietary intake and body weight, improved.

With regard to interventions with artificial nutrition by enteral/parenteral supplementation, interesting effects were demonstrated. Two studies compared enteral/parenteral supplementation with standard nutritional counseling alone in head and neck and gastrointestinal cancers on muscle strength; one study demonstrated deterioration of strength over time [43], while the other one showed an improvement [46]. In the first case, the advanced stage of the disease or its site (head and neck) may explain the subsequent negative findings. Similar positive results to those of Obling et al. [46] were demonstrated by another study, where the effects of optimization of oral intake by an individualized approach (including counseling, ONS, or artificial nutrition) alone was compared to that of an exercise program associated with the same nutritional care [47]. Only one uncontrolled study analyzed the effects of short-term (seven days) parenteral nutrition on body weight, body composition, and muscle strength, demonstrating clinical benefits in regard to all of these targets [44]. However, it should be noted that results from short-term studies cannot necessarily be extrapolated to long-term applications, since some of these effects may not persist, while others (such as the specific anabolic effect of proteins on muscle strength) may need longer periods to manifest. 

An interesting issue is the relationship between laboratory parameters, sarcopenia–cachexia, muscle strength, and nutritional interventions. Cachexia is characterized by the activation of systemic inflammation, which is reflected by increased serum levels of C-reactive protein [57]; however, the catabolic drive during cancer cachexia can also be reflected by other cytokines, including IL-6 and TNFα. Increased levels of these cytokines were reported in association with sarcopenia and cachexia during conditions such as aging [58,59] and cancer [60], and nutritional interventions targeted at suppressing inflammation were shown to ameliorate cancer cachexia in experimental models [61]. The effects of protein and amino acid supplementation alone or associated with other interventions on serum concentrations of proinflammatory cytokines were investigated in three studies [36,39,40]. Despite increased muscle strength and mass, all of the studies failed to show any effect of nutrition intervention on inflammatory cytokines after treatment, suggesting a poor correlation between muscle strength, lean mass, and circulatory markers of systemic inflammation. In contrast, a closer direct association between laboratory parameters of nutrition status (albumin and prealbumin), muscle strength, and dietary intervention was confirmed in most of the studies under consideration [36,41,44]. This finding was in line with the concordance between reduced handgrip strength and low albumin/prealbumin levels, among other factors, in malnourished cancer patients assessed using the Global Initiative for Malnutrition (GLIM) criteria for malnutrition diagnosis [28].

Another interesting point is the association between nutritional intervention, change in muscle mass and strength, and treatment-correlated outcomes. Three studies [35,41,45] included treatment tolerability and time to stem cell engraftment, among other endpoints, all showing concordance regarding an association between the positive effects of nutritional treatment on maintenance of muscle strength and improved treatment outcomes, despite non-significant changes in body mass index and reduced calorie and protein intake in one of the three studies [41]. Although encouraging, these findings need to be contextualized within the whole picture.

Overall, the results of these studies highlighted the difficulties present when extrapolating the specific effects of proteins/amino acids or their derivatives targeted at muscle mass recovery/maintenance on muscle strength. The first problem is related to the small number of data available regarding the relationship between muscle mass and strength in cancer patients. In spite of a linear correlation between the two reported by some authors [26], others failed to demonstrate it. Results from the latter studies instead showed preserved muscle function in the early stages of the disease, in spite of significant muscle loss and a prevalent decline during the late stage of cachexia prior to minor loss of muscle mass [62]. Thus, the heterogeneity of study populations in terms of cancer type and stage and baseline nutritional status could hamper conclusions when comparing the results of different trials. Another problem is related to the adequacy of calorie intake, which is essential to elicit the maximal stimulation of protein synthesis and to unveil the anabolic potential of proteins on muscle mass and strength. Because cancer patients often do not reach their energy targets, the selective effects of amino acid supplementation on muscle strength and mass could be blunted. A final issue is the paucity of data available regarding the effects of specific types of amino acids or their derivatives which stimulate protein synthesis and, subsequently, result in increased muscle mass and strength, as well as the lack of studies examining the interactions between protein supplementation and anabolic stimuli other than pharmacological therapies or resistance training. The effects of protein type and dose and co-ingestion of nutrients (especially in combination with a carbohydrate load, which stimulates insulin secretion) should be addressed in conditions of adequate energy requirement coverage. This could be done in populations treated exclusively by enteral/parenteral nutrition, in whom protein and calorie requirements and delivery can be easily calculated. 

### Limitations

The results of this review should be interpreted in light of some potential methodological limitations. Only two databases, although relevant, were used to perform the literature search. Moreover, despite a rigorous search and review process, some relevant manuscripts may not have been considered because of the choice of databases, the search strategy, and the method of article selection. In addition, a temporal window of ten years was selected and explored; we cannot exclude that expanding the search to a longer period of time could have led to the inclusion of additional material, which would have allowed us to draw further conclusions.

## 5. Summary and Conclusions

In summary, the available data demonstrate a concordance between changes in muscle mass and strength following ingestion of isolated proteins or amino acids, however the effects reported by different studies are discrepant. In contrast, after supplementation of energy and proteins via ONS/enteral/parenteral nutrition, the effect on muscle mass and strength appears to be more robust. Whether this result is the effect of the correction of the energy deficit or of an interaction between proteins and other macronutrients is not clear from the available evidence. Finally, other anabolic stimuli, such as physical exercise, seem to exert stronger effects on muscle strength than that of isolated amino/acid protein supplementation.

Data regarding clinically relevant issues, such as the relationship between muscle mass and strength after dietary protein supplementation in cancer patients losing weight, are lacking, but should be definitively established in larger, long-term, randomized controlled trials. Potential benefits of long-term protein/amino acid supplementation, especially at high doses, should be balanced against possible risks, such as renal problems related to primary disease and/or its treatment. Indeed, periodic re-evaluation of protein supplementation is mandatory to avoid deterioration of renal function in patients with tumors involving the urogenital tract or in those presenting recurrent problems related to treatment toxicity, such as dehydration. Evidence regarding the long-term efficacy and safety of protein and amino acid supplementation on muscle strength should be provided by future studies. 

## Figures and Tables

**Table 1:** Final strings used for searches run in the PubMed and Scopus databases.

Database	Search String
PubMed	((neoplasia* OR neoplasm* OR tumor OR tumors OR tumour OR tumours OR cancer OR cancers OR malignan*) NOT necrosis) AND supplement* AND (protein OR proteins OR “amino acid*” OR aminoacid* OR BCAA* OR “branched chain amino acid*” OR leucine OR methylbutyr* OR “carnitine” OR “arginine” OR “glutamine”) AND (asthenia OR fatigue OR “muscle strength*” OR “muscular strength*” OR “handgrip strength*” OR “hand grip strength*” OR “hand-grip strength*” OR “grip strength*” OR “muscle mass” OR ffm OR “fat free mass” OR “lean mass” OR “lean body mass” OR dynapaenia OR myopenia) AND “last 10 years”[PDat] Filters: English
SCOPUS	(TITLE-ABS-KEY (neoplasia* OR neoplasm* OR tumor OR tumors OR tumour OR tumours OR cancer OR cancers OR malignanc*) AND TITLE-ABS-KEY (supplement*) AND TITLE-ABS-KEY (protein OR proteins OR “amino acid*” OR aminoacid* OR bcaa* OR “branched chain amino acid*” OR leucine OR methylbutyr* OR “carnitine” OR “arginine” OR “glutamine”) AND TITLE-ABS-KEY (asthenia OR fatigue OR “muscle strength*” OR “muscular strength*” OR “handgrip strength*” OR “hand grip strength*” OR “hand-grip strength*” OR “grip strength*” OR “muscle mass” OR ffm OR “fat free mass” OR “lean mass” OR “lean body mass” OR dynapaenia OR myopenia) AND NOT TITLE-ABS-KEY (necrosis)) AND DOCTYPE (ar) AND PUBYEAR > 2009 AND (LIMIT-TO (LANGUAGE, “English”))

*: truncation symbol (“wildcard”) used to search all terms having a same root.

**Table 2:** Summary of main characteristics of the studies included in the review.

Country Author Year Published	Design Follow up time Days (d) Weeks (w) Months (m)	Population Male/Female (M/F) Age (y) Patient Characteristics Cancer type Treatment Modality	Nutritional Intervention	Endpoints	Main Results
Spain Arribas, 2017 [43]	Prospective Follow up: 3 m	*N* = 20 M/F: 19/1 Age: 53.7 ± 7.11 Outpatients Head and neck squamous carcinoma Chemoradiotherapy (CRT)	Dietetic counseling and nutritional supplementation according to the individual needs estimated by standard formulas. Protein requirement: 1.5 g/kg/d. Enteral nutrition (EN) by nasogastric tube (NGT) used in 35% of patients.	Changes in Patient Generated- Subjective Global Assessment (PG-SGA), body weight (BW), body mass index (BMI), muscle strength (MS), fat free mass (FFM), serum albumin, and energy and protein intake.	Significant decreases in BW, BMI, MS, and FFM; no significant changes in serum albumin, protein, or energy intake.
Italy Caccialanza, 2019 [44]	Single-arm clinical trial Follow up: 7 d	*N* = 118 M/F: 76/42 Age: 59.9 ± 14.7 Inpatients at nutritional risk with contraindications for EN Mixed tumors Chemotherapy (CT)/Radiotherapy (RT)/Palliative care	7-day supplemental parenteral nutrition (SPN) (glucose, amino acids, lipids, electrolytes, multivitamin, and multimineral elements) to integrate oral intake in order to meet calorie requirements estimated by standard formulas. Protein requirement: 1.5 g/kg/d.	Changes in phase angle (PhA), BW, BMI, MS, and prealbumin (PAB).	SPN resulted in significant improvements in PhA, BW, BMI, MS, and PAB.
Italy Cereda, 2018 [45]	Randomized controlled trial Follow up: 3 m after the end of RT	*N* = 159 M/F: 114/45 Age: 63.8 ± 12.7 (COUNS) 66.5 ± 14.5 COUNS+ONS) Outpatients Head and neck cancer (HNC) RT	Nutritional counseling (COUNS) with or without oral nutritional supplements (ONS) (250 mL/day of an oral formula containing 500 kcal, 23 g protein, 1.9 g omega-3 fatty acids). Calorie requirements estimated by Harris Benedict formula; protein requirement set at 1.2 g/kg/d.	Changes in BW, protein-calorie intake, MS, PhA, and quality of life (QoL); anticancer treatment tolerance.	In ONS group, minor BW loss, improved energy and protein intake, and QoL; trend toward significance for MS (*p* = 0.057); no significant differences for PhA. In ONS group, less (*p* = 0.029) need for changes in anticancer treatments due to toxicity.
Italy Cereda, 2019 [35]	Randomized controlled trial Follow up: 3 m	*N* = 166 M/F:100/66 Age: 65.7 ± 11.4 (COUNS) 65.1 ± 11.7 (COUNS+WPI) Malnourished patients Advanced mixed tumors Candidate to or undergoing CT	Nutritional counseling (COUNS) with or without whey protein isolate (WPI) supplementation (20 g/d). Calorie requirements estimated by Harris Benedict formula; protein requirement set at 1.5 g/kg/d.	Changes in PhA, standardized phase angle (SPhA),fat-free mass index (FFMI), BW, MS, and CT toxicity.	Significantly improved PhA, SPhA, FFMI, BW, and MS, reduced risk of CT toxicity in WPI as compared with COUNS.
US Dawson, 2018 [37]	Randomized controlled trial Follow up: 12 w	*N* = 37 M/F: 37/0 Age: 66.3 ± 9.0 (PRO and control), 68.6 ± 8.4 (TRAINPRO and TRAIN) Outpatients Prostate cancer Androgen deprivation therapy	Pts assigned to resistance training and protein supplementation (TRAINPRO), resistance training (TRAIN), protein supplementation (PRO), or control. TRAINPRO and PRO: 50 g/day of WPI.	Changes in lean mass (LM), appendicular skeletal muscle (ASM) index, body fat %, MS, physical function, QoL, metabolic syndrome (MetS) score, and MetS components.	Resistance training significantly increased LM, appendicular skeletal mass, and sarcopenic index, and decreased body fat %. No interaction effects of TRAIN and PRO for any outcome.
Denmark Lonbro, 2013 [38]	Randomized controlled trial Follow up: 2 m	*N* = 30 M/F: 23/7 Age: 56 (PROCR), 59 (PLA) Outpatients HNC Terminated RT ± CT ± Immunotherapy	Creatine (5 g) and protein (30 g) supplementation (PROCR) or placebo (PLA) in close relation to progressive resistance training session.	Changes over time and group differences in lean body mass (LBM), MS, and functional performance.	Significant LBM, MS, and functional performance increase in both groups. No significant group differences in any endpoints.
Italy Madeddu, 2010 [36]	Uncontrolled trial Follow up: 8 w	*N* = 25 M/F: 13/12 Age: 65.8 ± 11.4 Cachectic pts stage IV Mixed tumors at any site Active antineoplastic treatment	Oral amino acid functional cluster (AFC) containing 4 g of essential amino acids.	Changes in BW, BMI, LBM, MS, fatigue, and laboratory (albumin, fibrinogen, C-reactive protein (CRP), tumor necrosis alpha (TNFα), leptin, and reactive oxygen species (ROS)) variables.	Significant increases in MS and serum albumin and decrease (*p* = 0.001) in ROS levels. Trend toward increased body weight (*p* = 0.056) and leptin (*p* = 0.052). No significant changes in CRP, IL-6, or TNFα.
US Madzima, 2017 [39]	Cohort Follow up: 12 w	*N* = 33 M/F: 0/33 Age: 59 ± 8 Survivors Breast cancer Terminated treatment	Patients assigned to resistance training (RT) or RT + whey/casein protein isolate (W/CPI) supplementation (40/d).	Changes in MS, LBM, fat body mass (FBM), insulin growth factor 1 (IGF-1), adiponectin, and CRP.	Both groups significantly increased IGF-1, MS, and LBM, and decreased FBM. No difference between groups. No change in adiponectin or CRP.
Italy Mantovani, 2010 [40]	Randomized controlled trial Follow up: 4 m	*N* = 332 M/F: 181/151 Age: 61.5 ± 9.7 (arm 1) 60.6 ± 13.5 (arm 2) 62.8 ± 11.5 (arm 3) 62.4 ± 11.9 (arm 4) 62.4 ± 9.4 (arm 5) Cancer-related anorexia/cachexia syndrome (CACS) Advanced tumors at any site CT or hormone therapy (HT)	Patients assigned to five arms: 1) medroxyprogesterone (500 mg/d) or megestrol acetate (320 mg/d); 2) oral high calorie and protein supplementation with eicosapentaenoic acid (EPA) (2.2 g/d); 3) L-carnitine 4 g/d; 4) thalidomide (200 mg/d); 5) combination of the above.	Change in LBM, resting energy expenditure (REE), fatigue, MS, appetite, proinflammatory cytokines, total energy expenditure (TEE), active energy expenditure (AEE), appetite, QoL, and Glasgow prognostic score (GPS).	In arm 5, LBM, REE, AEE, and MS were all significantly increased. Fatigue, GPS and Eastern Cooperative Oncology Group-Performance Status (ECOG-PS) were decreased. Appetite, IL-6, and TNFα were unchanged.
Denmark Obling, 2019 [46]	Randomized controlled trial Follow up: 24 w	*N* = 47 M/F: 20/27 Age: 66.9 (41.5–88.2) Outpatients at nutritional risk Gastrointestinal cancers Candidate for or undergoing CT	Patients assigned to two groups: 1) nutritional care and dietetic counselling; 2) nutritional care, dietetic counseling, and supplemental home parenteral nutrition (PN). Estimated requirements: energy 125 kJ/kg, protein 1.5 g/kg/d.	Changes in FFM, MS, QoL, and survival.	FFM and QoL increase in intervention group. MS increase in both groups, no difference between groups. No difference in survival.
China Ren, 2017 [41]	Randomized controlled trial Follow up: 30 d (pre transplantation) 30 d (post transplantation)	*N* = 24 M/F: 16/8 Age: 29.2 ± 18 (BP group) 31.6 ± 12 (ND group) Outpatients Acute leukemia Bone marrow transplantation	Natural diet + soy-whey protein blend (50% protein from whey and 50% from soy protein isolate) (BP) compared with natural diet (ND). Calorie and protein targets set at 35/kcal/kg/d and 1.5 g/kg/d in both groups.	Changes in BMI, upper arm muscle circumference (AMC), MS, serum albumin, time to stem cell engrafment.	In BP group, significant increases in AMC, MS, and serum albumin. Significantly shorter time to stem cell engrafment in BP.
Germany Schink, 2018 [47]	Non-randomized controlled trial Follow up: 12 w	*N* = 82 M/F: 74/57 Age: 59.9 ± 12.7 Advanced solid tumors at any site CT/RT/HT/other	Whole-body electromyostimulation (WB-EMS) physical exercise program twice a week vs CON (no exercise). In both groups, individualized nutrition counseling (energy intake ≥25 kcal/kg/die, protein: intake >1 g/kg/die), protein/amino acid-rich oral supplements, or EN or PN.	Change in SMM, body composition, BW, MS, QoL, fatigue, albumin, CRP	In WB-EMS group, significantly higher SMM and BW, improved physical function, and performance status. No significant differences in QoL, fatigue, albumin, or CRP. MS increased similarly in both groups.
Switzerland Uster, 2013 [48]	Randomized controlled trial Follow up: 3 m	*N* = 58 M/F: 46/12 Age: 63.8 ± 13.3 (NT) 66.2 ± 8.9 (CON) Outpatients, undernourished, or at high risk for malnutrition Mixed tumors at any site	Individualized nutritional intervention (counseling + food fortification and ONS if required (NT)) versus no intervention (CON).	Changes in dietary intake, BW, performance status, MS, and QoL.	In intervention group, significantly higher energy and protein intake. No significant improvements in nutritional status, MS, physical functioning, or QoL.
Japan Wada, 2018 [42]	Randomized controlled trial Follow up: 3 d pre-operatively 7 d post-operatively	*N* = 60 M/F: 34/26 Age: 66 (40–681) (NT); 69 (25–81) (CON) Inpatients Abdominal cancers Surgery	NT: Beta-hydroxy-beta-methylbutyrate (HMB) (1.2 g)/arginine (Arg) (7 g)/glutamine (Gln) (7 g) once daily preop and postop. CON: Equivalent amount of isocaloric juice.	Wound complications, length of hospital stay (LOS), skeletal muscle mass (SMM), MS, and skin water content.	No significant differences between groups for any of the explored endpoints.
